# Robot-assisted endoscopic third ventriculostomy under intraoperative CT imaging guidance

**DOI:** 10.1007/s00701-023-05713-4

**Published:** 2023-07-25

**Authors:** Angela Elia, Luca Paun, Johan Pallud, Marc Zanello

**Affiliations:** 1 Service de Neurochirurgie, GHU Paris Psychiatrie et Neurosciences, Site Sainte Anne, 1, rue Cabanis, F-75014 Paris, France; 2 Université Paris Cité, Paris, France; 3 Institute of Psychiatry and Neuroscience of Paris, INSERM U1266, F-75014 Paris, France

**Keywords:** Computer assisted, Hydrocephalus, Image processing, Intraoperative monitoring, Robotic surgical procedures

## Abstract

**Background:**

The robot-assisted neurosurgical procedures have recently benefited of the evolution of intraoperative imaging, including mobile CT unit available in the operating room. This facilitated use paved the way to perform more neurosurgical procedures under robotic assistance. Endoscopic third ventriculocisternostomy requires both a safe transcortical trajectory and a smooth manipulation.

**Method:**

We describe our technique of robot-assisted endoscopic third ventriculocisternostomy combining robotic assistance and intraoperative CT imaging.

**Conclusion:**

Robot-assisted endoscopic third ventriculocisternostomy using modern intraoperative neuroimaging can be easily implemented and prevented erroneous trajectory and abrupt endoscopic movements, reducing surgically induced brain damages.

**Supplementary Information:**

The online version contains supplementary material available at 10.1007/s00701-023-05713-4.

## Relevant surgical anatomy

**Fig. 1:**
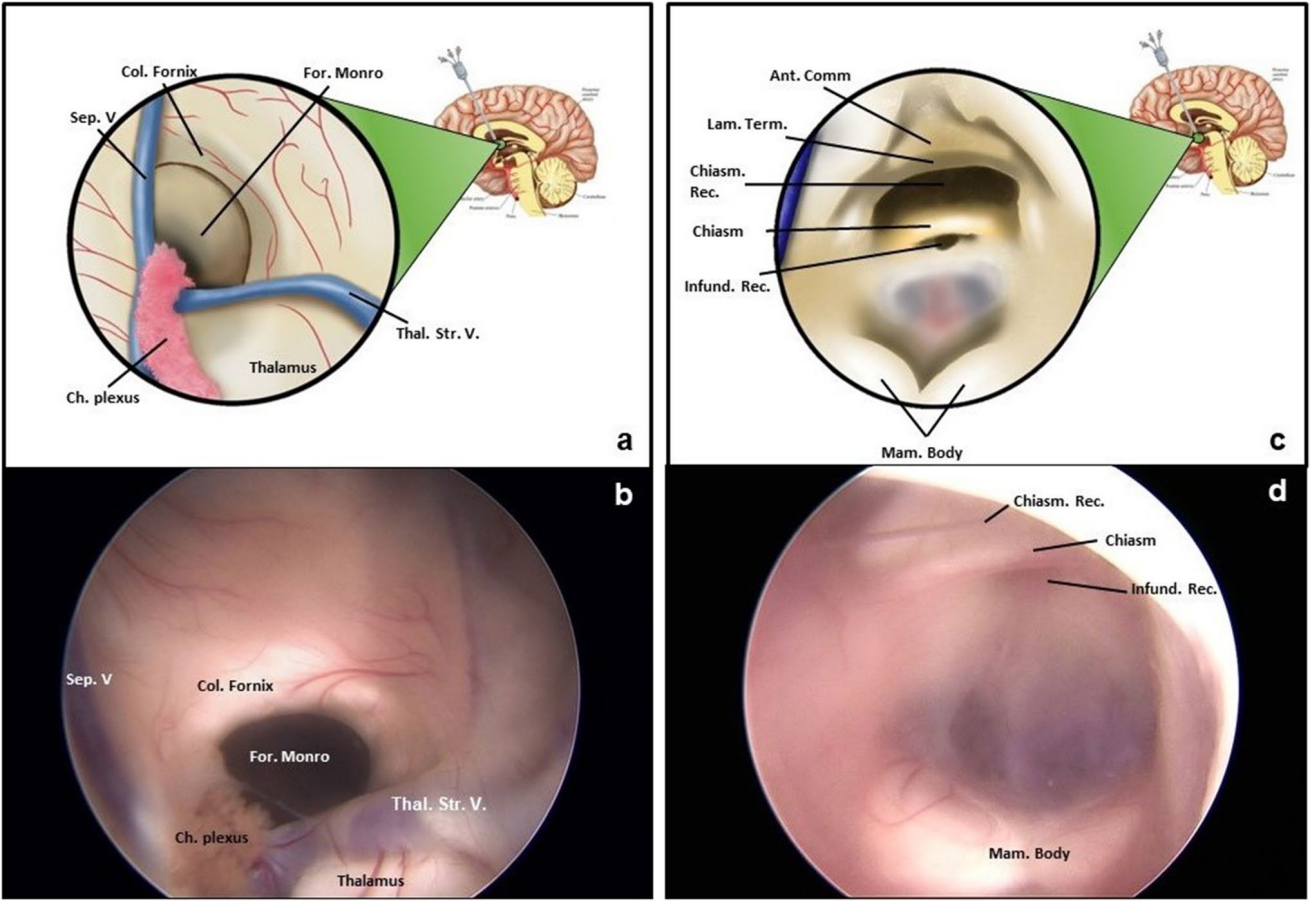
The relevant endoscopic anatomy of the third ventricle. **a** Schematic illustration of endoscopic anatomy of the interventricular foramen of Monro: on the medial wall the septal vein crosses with the choroid plexus while the thalamo-striate vein should be visualized laterally. At the angle of these two veins, the interventricular foramen should be visualized. It is delimited anteriorly by the anterior pilar of the fornix, posteriorly by the anterior pole of the thalamus, and inferiorly by the inter-thalamic adhesion. **b** Intraoperative endoscopic view of the anatomy of the interventricular foramen of Monro that is located at the junction between the anterior wall and the roof of the third ventricle. **c** Schematic illustration of endoscopic anatomy of the anterior wall, extending from the interventricular foramen of Monro to the optic chiasm, and the floor of third ventricle, extending from the optic chiasm to the cerebral aqueduct. From anterior to posterior, the anatomical landmarks are the anterior commissure, the lamina terminalis, the optic chiasm, the pituitary stalk defined by the infundibular recess, and the mamillary bodies. **d** Intraoperative endoscopic view of the anatomy of the floor of the third ventricle: in the most anterior part there is a prominence formed by the optic chiasm; just below the optic chiasm the third ventricle extends into the pituitary stalk, forming the infundibular recess. In the most posterior the mamillary bodies are visualized as a round and paired prominence

The proper knowledge of the anatomy of the lateral and third cerebral ventricles is essential. When the lateral ventricle is reached with the endoscope, the choroid plexus represents the first and the easiest identifiable landmark. Laterally to the choroid plexus, the thalamo-striate vein can be visualized while the septal vein can be visualized medially. The interventricular foramen of Monro is located at the angle of these two veins. It is delimited anteriorly by the anterior pilar of the fornix, posteriorly by the anterior pole of the thalamus, and inferiorly by the inter-thalamic adhesion (Fig. 1a and b). In cases of chronic hydrocephalus, spontaneous fenestrations of the septum pellucidum (usually thinner than usual) can be present and should not be misinterpreted as the interventricular foramen.

When crossing the interventricular foramen with the endoscope, the floor of the third ventricle is visualized, extending from the anterior commissure to the mammillary bodies (Fig. 1c and d). The floor is delimited anteriorly and rostrally by the lamina terminalis; medially by the chiasmatic recess, the optic chiasm, and the infundibular recess (following an anterior-posterior disposition); and posteriorly by the tuber cinereum and the mammillary bodies. In case of chronic hydrocephalus, the floor of the third ventricle can be thinner than usual.

## Description of the surgical technique

Endoscopic third ventriculostomy still carries some risks in modern series [2, 4]. A way to reduce complications is to ensure a safe trajectory and a smooth displacement of surgical tools into the patient’s brain. Robotic assistance has already been explored but could be challenging to set up [6, 12]. The advent of 3D intraoperative imaging tool and new frameless registration should overcome this pitfall.

## Preoperative planning

**Fig. 2:**
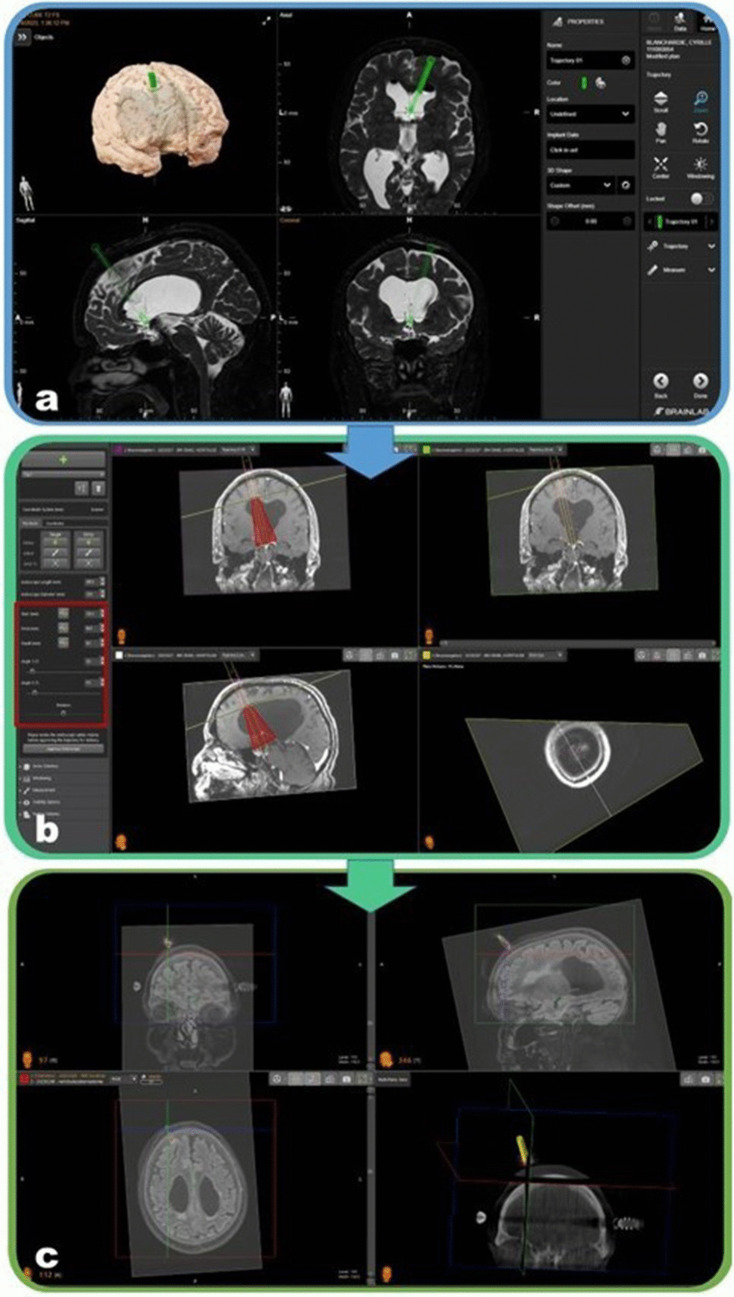
The preoperative planning of endoscopic third ventriculocisternostomy trajectory. **a** Using the BrainLAB Elements software, tridimensional gadolinium-T1- and T2-weighted sequences were used to define the most suitable trajectory defined as the shortest perpendicular pathway from right frontal gyrus to the interventricular foramen of Monro avoiding cerebral sulci and vessels. **b** The coordinates of the trajectory are transferred to the NeuroInspire software and endoscopic parameters are added: endoscopic outer diameter is set at 6 mm, while the endoscopic length is 150 mm. At this point, the workspace and the volume of movement of endoscope are defined (*red triangles*): the pivot point is placed at the cortico-ventricular junction, the depth is placed just below the floor of the third ventricle, and the degrees of freedom in the two axes (antero-posterior and latero-later angle) are chosen according to anatomical characteristics (*red square*). **c** An intraoperative tridimensional scan is acquired with cone beam O-Arm and co-registered with preoperative MRI through the Neuroinspire software to check the correct location of the robot along the axis of the planned endoscopic third ventriculocisternostomy trajectory using a Kirschner wire

Preoperatively, a straight trans-cerebral trajectory (with a diameter equal to the endoscope diameter plus a 2 mm margin) is planned on preoperative MRI using tridimensional T1-weighted contrast-enhanced and T2-weighted sequences for vascular and anatomical assessment [1]. The sequences are co-registered using the BrainLAB Elements software (BrainLAB AG, Feldkirchen, Germany) (Fig. 2a). The entry point is placed onto or near the coronal suture. The planned trajectory crosses the cortex perpendicularly and stretches from the right frontal pole, usually the superior or middle frontal gyrus, to the interventricular foramen, avoiding sulci and cortical vessels. The trajectory coordinates are then transferred into the NeuroInspire software (Renishaw, New Mills, UK) and the endoscopic parameters (starting point, pivot point, degrees of freedom) are calculated. The entry point is placed at the cortical surface; the pivot point is placed at the cortico-ventricular junction. The degrees of freedom for the endoscopic movement along the two axes (antero-posterior and latero-medial angle) are chosen according to anatomical characteristics, usually 15°. This step defines the safety area for endoscopic navigation (Fig. 2b).

## Patient position and intraoperative planning

**Fig. 3:**
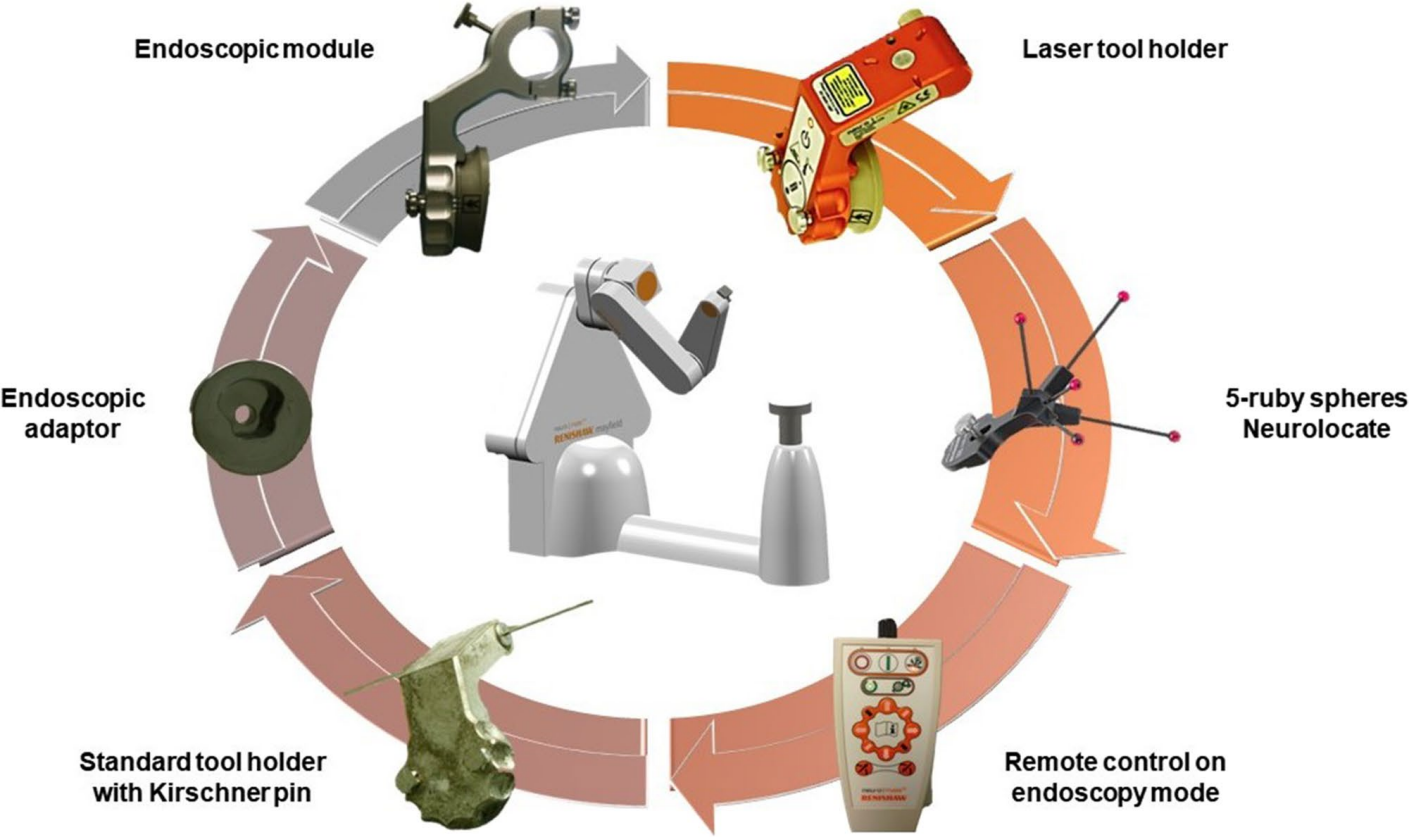
Overview of all robot tools used during a robot-assisted endoscopic third ventriculocisternostomy. According to the timeline from above to below, the laser tool holder used to support the Neurolocate tool and to check the correct position of the robot arm; the five-ruby spheres Neurolocate tool that plays as fiducials markers for the frameless patient registration method; the remote control with the endoscopy mode to drive the robot arm and the endoscope; the standard tool holder used to fix the metallic pin during the endoscopic third ventriculocisternostomy trajectory check; the endoscopic adaptor to fix the endoscope on the endoscopic module

Robot-assisted endoscopic third ventriculocisternostomy is performed under general anesthesia. Patient’s position and intraoperative planning procedure are similar to the robot-assisted stereotactic biopsy previously reported [3, 11]. In dorsal decubitus, the patient’s head is fixed on Talairach head holder (Dixi, Besançon, France) but a wide variety of frame can be used, including Leksell G base frame or the head clamp solution provided by Renishaw. The five-ruby spheres Neurolocate™ registration module is centered on the vertex of patient’s head. A tridimensional scan is acquired with the cone beam O-Arm (Medtronic, Minneapolis, MN) and then co-registered with preoperative MRI-based planning through the NeuroInspire software (Fig. 2c). Using a wire metallic pin, the endoscopic third ventriculocisternostomy trajectory is checked using a second tridimensional scan acquisition [10, 11]. The endoscopic tool holder is then installed, and the remote control is settled on endoscopic navigation to intraoperatively navigate the robotic arm (Fig. 3).

## Surgical technique

A 3-cm-long linear skin incision centered on the entry point is performed. A burr hole is performed with a 14-mm-diameter cranial bone drill. After coagulation, the dura mater is opened. The endoscope is placed on the endoscopic module of the robot arm and slowly navigated along the trajectory using the remote control. We used a Minop trocar (BBraun, Melsungen, Germany) with an outer diameter of 6 mm, a length of 150 mm, and a 30° angle. Under direct visual control, the endoscope is moved throughout the lateral ventricle and the interventricular foramen to reach the third ventricle. The target site for the fenestration is just anterior the mamillary bodies on the midline. After potential minimal coagulation, a fenestration is performed with endoscopic scissor. Then, it is enlarged using the NeuroBalloon™ Catheter (Integra LifeSciences). The black mark defines the distal limit of catheter introduction. The double balloon is slowly inflated with 0.6 to 1.0 ml of air asymmetrically: the proximal balloon is inflated first to keep the catheter in the correct position, and then the distal one is inflated to anchor the membrane. Last, the whole double balloon is inflated to complete the fenestration with the balloon waist. An adequate fenestration should provide a good access into the prepontine cistern and a clear visualization of the basilar artery. At the end of the procedure, the endoscope is gently removed, and the skin incision is sutured.

## Indications

**Fig. 4:**
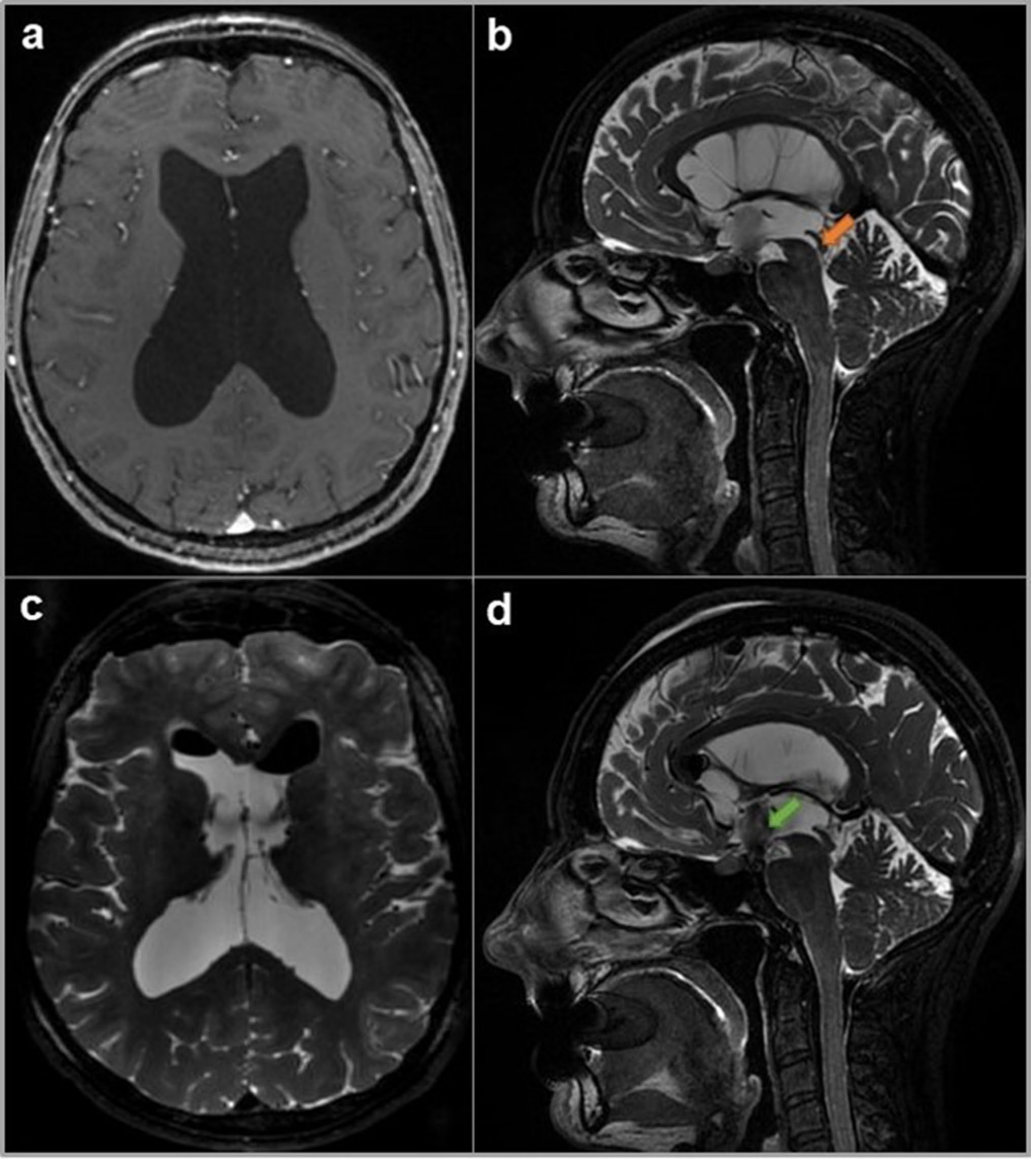
Pre- and post-operative MRI of an obstructive hydrocephalus treated by a combined CT imaging and robot-assistance endoscopic third ventriculocisternostomy. **a** Pre-operative axial gadolinium T1-weighted MRI revealed an enlargement of the lateral ventricles and a tri-ventricular hydrocephalus. **b** Pre-operative sagittal T2-weighted MRI demonstrate a superiorly narrowing of the aqueduct (*orange arrow*) with a fourth ventricle not dilated, o note the absence of flow-void signal intensity at the aqueductal level and the absence of trans-ependymal edema. **c** Post-operative axial T2-weighted MRI demonstrate a progressive reduction in ventricular size. **d** Post-operative sagittal T2-weighted MRI confirmed the correct fenestration in the floor of third ventricle and excellent flow passing through the ventriculostomy (*green arrow*)

The main indication of the robot-assisted stereotactic endoscopic third ventriculocisternostomy is obstructive hydrocephalus (Fig. 4). The different etiologies of non-communicating hydrocephalus are congenital aqueductal stenosis, posterior third ventricle tumor, tectal tumor, tumor compressing the cerebral aqueduct, intraventricular hematoma, cerebellar infarct, Dandy-Walker malformation, vein of Galen aneurism, and syringomyelia with or without Chiari malformation type I [9, 10]. Endoscopic third ventriculocisternostomy can be discussed in front of normal pressure hydrocephalus or idiopathic normal pressure hydrocephalus [5, 8].

Robot-assisted stereotactic intraventricular endoscopy with intraoperative CT imaging can be also used for tumor removal, using other endoscopic tools ).

## Limitations

Comparing to classical endoscopic third ventriculocisternostomy, the robot-assisted technique lasts longer initially due to the need for several 3D acquisitions and co-registrations into the robot system. However, if the first procedures could last about 2 h, the length of the procedure decreased quickly to 1 h, similar to the freehand surgical procedure. Moreover, the application of the robot combined with the intraoperative CT requires an experienced surgical team, ideally already familiar with robot-assisted procedure. Lastly, robot-assisted endoscopic third ventriculocisternostomy uses high technological and expensive devices.

## How to avoid complications

An attentive preoperative planning of the safest endoscopic third ventriculocisternostomy trajectory based on the analysis of individual patient’s anatomy including vessels, sulci, gyri, and intraventricular structures is essential to avoid complications as severe hemorrhage, memory loss, hemiparesis, and hypothalamic dysfunction [2]. If possible, the trajectory should be reviewed by two neurosurgeons at the eve of the surgery. A recent MRI, classically acquired in the week preceding the surgery, is recommended.

All endoscopic instruments should be always in the field of view of the endoscopic camera to ensure a constant direct visual control and to avoid accidental neural or vascular injuries.

Last, the identification of intraventricular anatomical landmarks is a key point to properly orientate and navigate the endoscopy and to avoid erroneous assumptions.

## Specific information for the patient

Patient should be informed about the specific endoscopic third ventriculocisternostomy-related complications, including not only intraventricular hemorrhage and intracranial hematoma, infections, and cerebrospinal fluid leak with a reported incidence rate ranging between 0.8 and 3.7% but also hemiparesis, gaze palsy, memory disorders, altered consciousness, diabetes insipidus due to thalamic, forniceal, hypothalamic, and midbrain injuries in 0.9–1.5% of cases, and death [2].

Another essential information for the patient is the possibility of endoscopic third ventriculocisternostomy failure. Indeed, the reported success rate of endoscopic third ventriculocisternostomy ranges between 60 and 91.5% and it is related to the etiology of the hydrocephalus with the highest failure rate in cases of previous shunt surgery and complex hydrocephalus, and the lowest in cases of obstructive hydrocephalus secondary to aqueduct stenosis or tumor [7].

### Supplementary information

ESM 1 (MP4 67898 kb)
